# Supplementary material for: DMRforPairs: identifying Differentially Methylated Regions between unique samples using array based methylation profiles
Source: BMC Bioinformatics. 2014 May 15;15:141. doi: 10.1186/1471-2105-15-141 (PMC4046028; doi:10.1186/1471-2105-15-141)
Supplement: Additional file 2 — DMRforPairs output for the comparison of A431-MCF7 and NA17018-NA17105. Please start from the HTML files in each folder. Available via the BMC Bioinformatics website. [file 1471-2105-15-141-S2.zip › 1394847754114233_MOESM2_ESM/A431_MCF7/figures/22017.pdf]

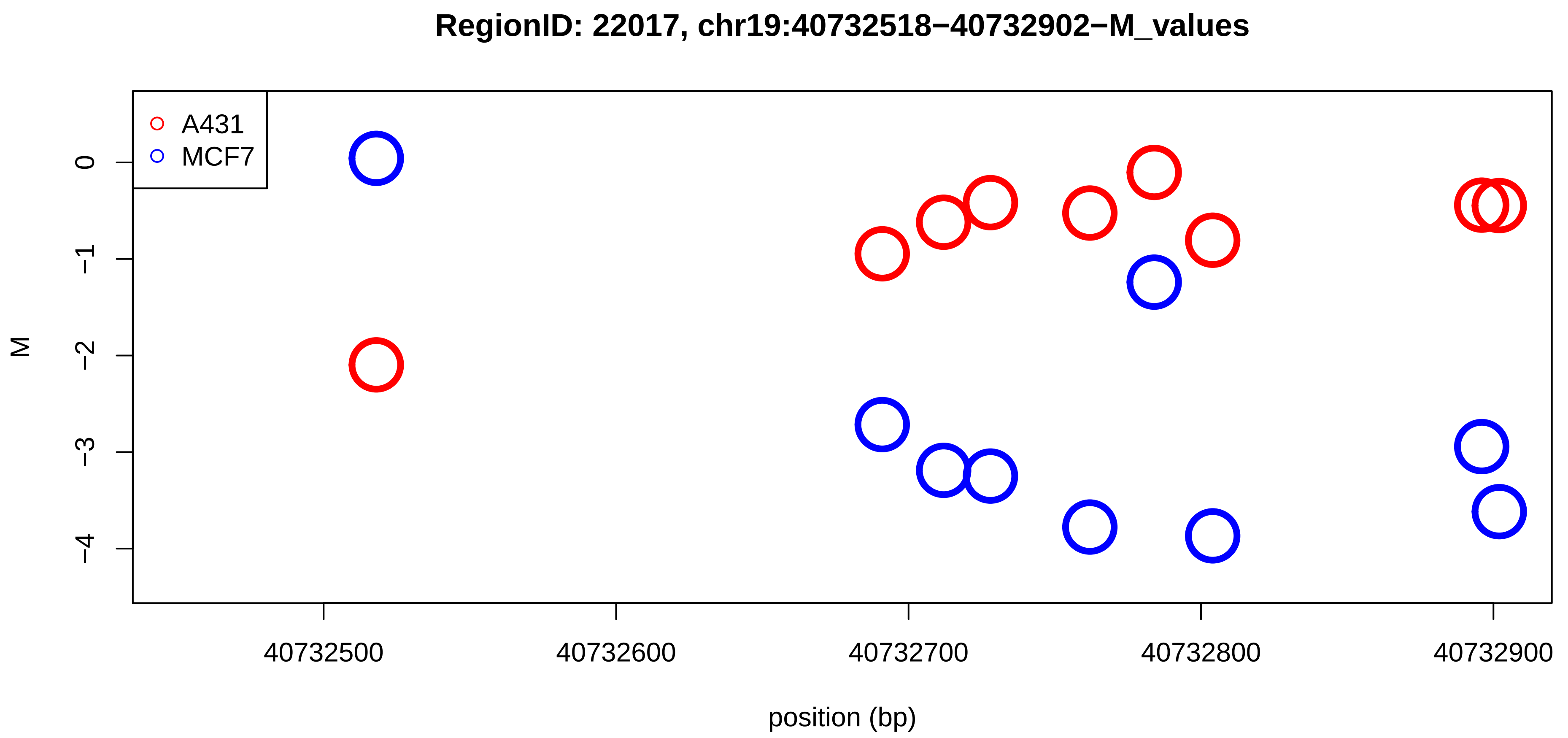

RegionID: 22017, chr19:40732518−40732902−M_values
A431
MCF7
M
0
−1
−2
−3
−4
40732500
40732600
40732700
40732800
40732900
position (bp)

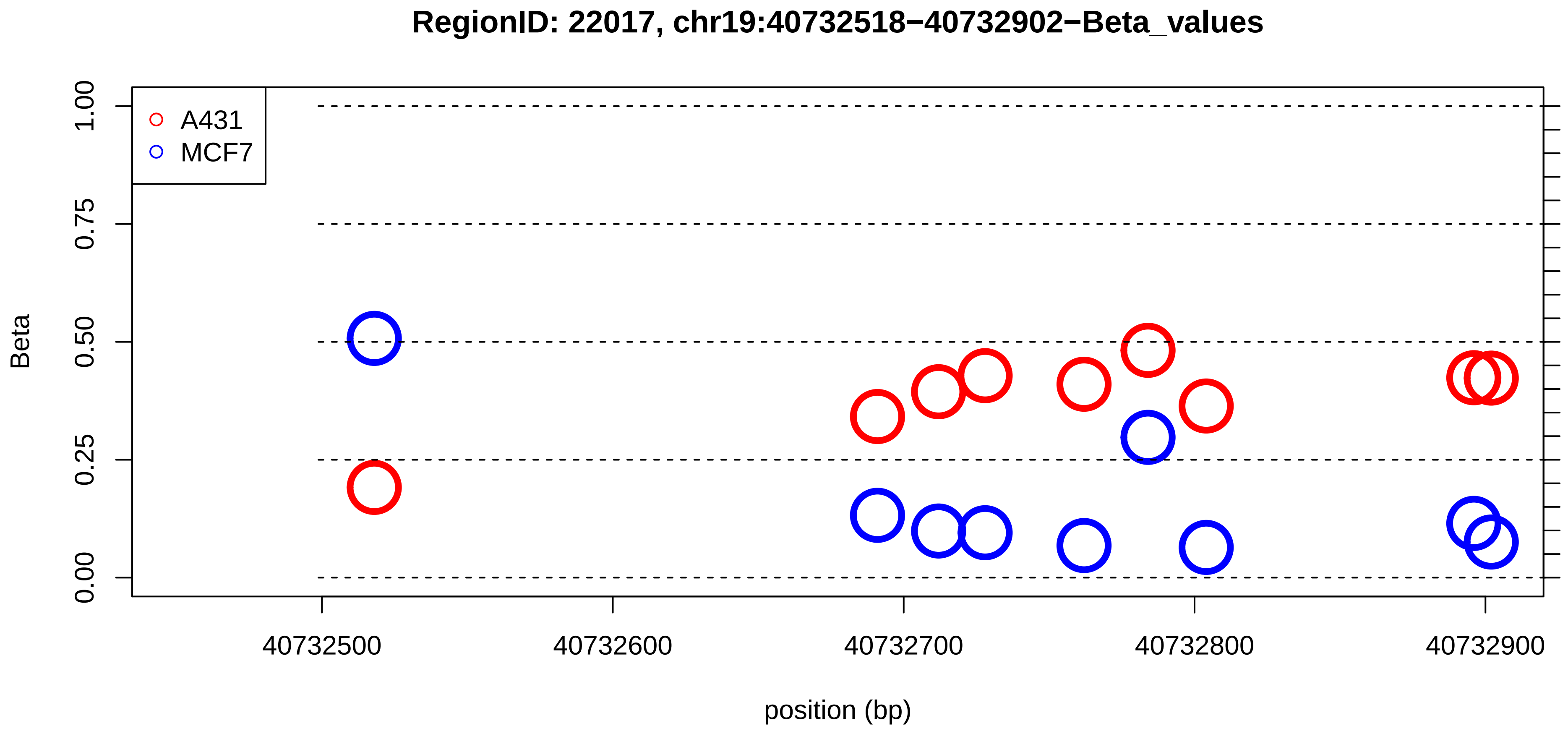

RegionID: 22017, chr19:40732518−40732902−Beta_values
A431
MCF7
Beta
1.00
0.75
0.50
0.25
0.00
40732500
40732600
40732700
40732800
40732900
position (bp)

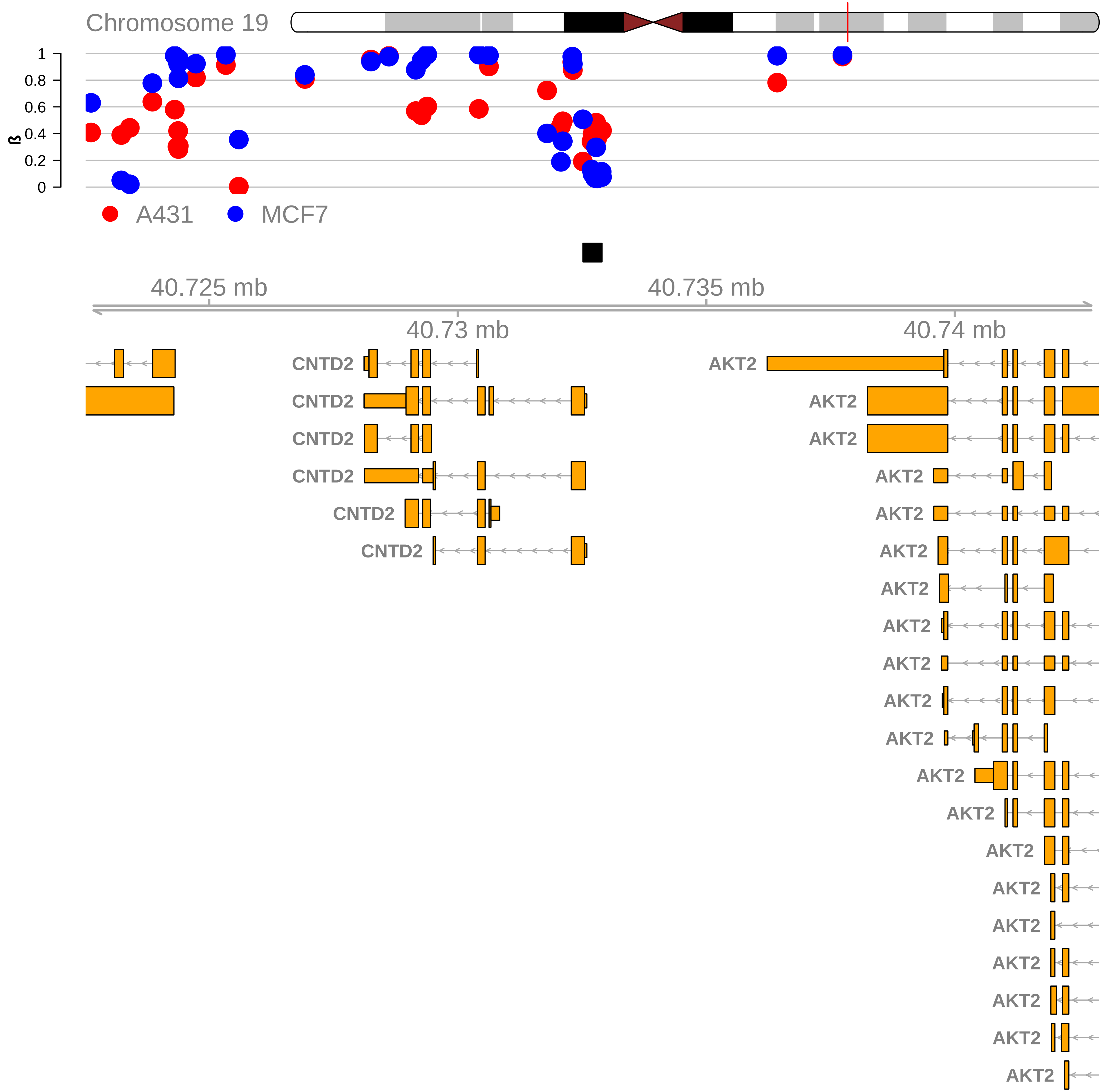
Chromosome 19
ß
1
0.8
0.6
0.4
0.2
0
A431
MCF7
40.725 mb
40.735 mb
40.73 mb
40.74 mb
CNTD2
CNTD2
CNTD2
CNTD2
CNTD2
CNTD2
AKT2
